# Semantics, Syntax or Neither? A Case for Resolution in the Interpretation of N500 and P600 Responses to Harmonic Incongruities

**DOI:** 10.1371/journal.pone.0076600

**Published:** 2013-11-05

**Authors:** Cara R. Featherstone, Catriona M. Morrison, Mitch G. Waterman, Lucy J. MacGregor

**Affiliations:** 1 Institute of Psychological Sciences, University of Leeds, Leeds, United Kingdom; 2 Medical Research Council, Cognition and Brain Sciences Unit, Cambridge, United Kingdom; Baycrest Hospital, Canada

## Abstract

The processing of notes and chords which are harmonically incongruous with their context has been shown to elicit two distinct late ERP effects. These effects strongly resemble two effects associated with the processing of linguistic incongruities: a P600, resembling a typical response to syntactic incongruities in language, and an N500, evocative of the N400, which is typically elicited in response to semantic incongruities in language. Despite the robustness of these two patterns in the musical incongruity literature, no consensus has yet been reached as to the reasons for the existence of two distinct responses to harmonic incongruities. This study was the first to use behavioural and ERP data to test two possible explanations for the existence of these two patterns: the musicianship of listeners, and the resolved or unresolved nature of the harmonic incongruities. Results showed that harmonically incongruous notes and chords elicited a late positivity similar to the P600 when they were embedded within sequences which started and ended in the same key (harmonically resolved). The notes and chords which indicated that there would be no return to the original key (leaving the piece harmonically unresolved) were associated with a further P600 in musicians, but with a negativity resembling the N500 in non-musicians. We suggest that the late positivity reflects the conscious perception of a specific element as being incongruous with its context and the efforts of musicians to integrate the harmonic incongruity into its local context as a result of their analytic listening style, while the late negativity reflects the detection of the absence of resolution in non-musicians as a result of their holistic listening style.

## Introduction

Studies exploring similarities between music and language have emphasised the fact that both music and language are combinatorial: their processing requires the integration of small units into structured wholes according to specific rules or probabilities [1]–[4]. The combinatorial nature of music and language means that, in both domains, rules and probabilities can be broken, either by error, or for effect. Studies focussing on neurocognitive processing across these two domains have shown that elements which are incongruous with expectations in music and language lead to strikingly similar neurophysiological responses [1], [3], [5]–[8]. These studies have also demonstrated shared resources in the processing of musical and linguistic incongruities [9], [10] and shared neural areas underlying the processing of complex music and language [11]–[18], although the extent to which these overlaps in neural activity reflect purely musical and linguistic processes rather than higher order processes at work in both domains, such as working memory and cognitive control, is under debate [19]. The fact that music and language both involve encoding, storing and integrating new information into a wider context according to rules and expectations which can be created and broken makes them invaluable tools for gaining insight into attention and working memory [1], [4], pattern processing, timing and sequence learning [4], [20], and transfer effects between different domains of human cognition [20]–[22]. Despite this recognition of the insights to be gained by studying how incongruities are processed in music and language, and despite the wealth of studies demonstrating similar late ERP components associated with musical and linguistic incongruities [1], [3], [5]–[8], the functional significance of the shared neurophysiological responses elicited by rule-bending words and notes is yet to be determined, in part because of the observation of two distinct late ERP effects elicited by harmonic incongruities: the P600 and the N500. The present study explores two possible explanations for the existence of these two different patterns, focusing on the harmonic resolution of the stimuli and the musicianship of listeners.

### What do we mean by “incongruity”?

In Western tonal harmony, notes in a musical piece are typically organised around a central key. This key (e.g. C Major) centres on a particular note (e.g. C), and determines which notes listeners can expect to hear (e.g. C, D, E, F, G, A, B), which combinations of notes are most likely to occur (e.g. the chords C–E–G, F-A–C, G-B–D), and which notes are likely to sound unexpected or even wrong to the listener (e.g. notes which do not belong in the key of C, such as F# and C#). Notes such as these, and chords which include these notes, have typically been referred to as “harmonic incongruities”.

Harmonic incongruities have repeatedly been shown in the literature to elicit two different ERP effects relative to harmonically congruous notes and chords: a positivity around 600 ms after the onset of the incongruity, or a negativity around 500 ms after the onset of the incongruity. These two ERP effects strongly resemble two well established effects from the field of psycholinguistics: a positivity around 600 ms after the onset of a syntactically unexpected word (e.g. the horse ran past the barn *fell), and a negativity around 400 ms after the onset of a semantically unexpected word (e.g. the man buttered his bread with his *socks). The similarities between these effects across music and language have led authors to equate harmonic processing either with the processing of linguistic syntax, or with the processing of linguistic semantics. However, no satisfactory explanation has been given for the existence of two distinct ERP effects associated with the processing of what authors commonly refer to as harmonic incongruities.

### Two distinct responses to harmonic incongruities in the literature

#### A late negativity

Following the establishment of the N400 ERP component elicited in response to semantic incongruities in language [23], music psychologists attempted to replicate this effect in response to harmonic incongruities in music. The determination to find this effect was underpinned by the shared combinatorial nature of music and language, and by the evidence for recruitment of overlapping neural resources in the processing of music and language.

Initial attempts were reported as unsuccessful [24]–[26]. However, a closer look at the figures reported by Hantz, Kreilick, Kananen, and Swartz (1997) suggested a relative negativity in the harmonically “open” ended sequences compared to the harmonically “closed” sequences. Hantz et al. [25] manipulated what they referred to as the harmonic closure of the musical sequences, in such a way that they either sounded finished or unfinished. As an example, if when singing the melody of “Mary had a little lamb” a singer did not go down by one note on the last word “school” but stayed on the same note as “to”, the melody would not sound complete, as the cadence which is set up by the preceding notes is left unresolved. Though the authors focussed on early ERP effects and on the patterns elicited by different conditions rather than on relative negativities or positivities shown when comparing conditions, the data they report suggests a relative sustained negativity in the “open diatonic” condition (a chord which does not belong to the key of the piece and leaves the piece sounding unfinished) compared to the “closed” condition (where the piece ended as expected) in harmonised musical sequences. This negativity appeared to be maximal around 500 ms after the onset of the “open” final chord.

More recently, the N500, reviewed and interpreted by Koelsch (2011) as an indicator of musical semantics, has been widely adopted as a marker of harmonic incongruity processing. This effect is now a robust finding in the study of harmonic incongruity processing and has been described as a late negative component with an onset around 380 or 400 ms after the chord of interest, peaking around 550 or 570 ms [5], [27]. It has repeatedly been demonstrated to be elicited by harmonically incongruous chords, when the incongruous chord is the final chord in a chord sequence [5], [28]. The chords in these sequences are juxtaposed in such a way that the listener perceives a logical progression sounding like a harmonised melody, which leads to implicit expectations as to what the final chord will be. These sequence-final incongruities leave the sequences sounding unfinished or “harmonically unresolved” in such a way that the “unresolved” feel of these sequences is evident even to listeners with no musical training, adults and children alike [28]. The N500 elicited by these types of harmonic incongruities has been shown to be dependent on the build-up of harmonic context and to be elicited by melodic incongruities (incongruous notes within a tune) as well as harmonically incongruous chords [7].

In view of the similarities between this effect and the N400 elicited by semantic incongruities, researchers have set out to establish whether harmonic and semantic processing share neural resources, in an attempt to define the functional significance of the N500. Evidence in support of the interpretation of the N500 as an indicator of musical semantic processing comes from studies demonstrating interference effects. A study in which participants listened concurrently to harmonic sequences ending with an incongruous chord and sentences ending with an unexpected word, demonstrated that the N500 was not elicited by harmonic incongruities if participants were asked to focus on language rather than the music [29]. Semantic processing was also found to interfere with the ERP effects associated with harmonic processing when sentences were sung in a chorale-type phrase [30]. Steinbeis and Koelsch (2008) later demonstrated an interference of the semantic cloze-probability of words, presented concurrently with a harmonically incongruous chord, on the amplitude of the associated N500; this study also showed that the concurrent processing of syntactic incongruities had no effect on the amplitude of the N500. This finding was taken as evidence for the specificity of the harmony/semantics interference.

#### A late positivity

Before the establishment of the N500 as a marker of harmonic processing, a very different effect of harmonic incongruities was reported in an influential study by Patel, Gibson, Ratner, Besson, and Holcomb (1998). Patel et al. (1998) demonstrated a late positivity in response to incongruous chords compared to congruous chords in musicially trained listeners. The incongruous chords used in Patel et al.'s study were contained within an otherwise congruous sequence, and did not disrupt the feeling that the sequence had finished well: after the incongruous chord, which was borrowed from a different key, the piece returned to its original key, rendering the sequence “harmonically resolved”. This effect, identified as a P600 effect, was statistically indistinguishable from the P600 elicited by words which do not seem to fit within the syntactic structure of a sentence because of syntactically difficult embedded relative clauses (e.g. “endorsed” in “Some of the senators promoted endorsed* an old idea of justice”) [3].

Music-elicited positivities, often referred to as a Late Positive Component or LPC, have been found in response to incongruous elements in musical melody [31]–[33] and rhythm [31], [34], and have been shown to be stronger as participants' familiarity with the musical sequence increases, and in particiants with musical expertise [8], [34]. Its similarity to the P600 elicited by syntactically incongruous words in language led to the suggestion of an overlap between the cognitive mechanisms involved in processing linguistic syntax and harmony.

Slevc, Rosenberg, and Patel (2009) investigated interference effects between syntactic processing in language and harmonic processing in music in a self-paced reading task, using a similar approach to the previously discussed study by Steinbeis and Koelsch (2008). Each section of the sentence, typically one or two words long, was accompanied by a chord from a chorale-type sequence to allow the pairing of linguistic syntactic incongruities or semantic incongruities with musical harmonic incongruities embedded within harmonically resolved sequences. The results showed that the presence of a harmonic incongruity led to longer reading times on syntactically difficult words, but no change was seen in the reading times of semantically difficult words. This was taken as evidence of the specificity of the harmony/syntax processing interference.

### What underpins the existence of two distinct effects?

The studies discussed above present two contradictory stories. On the one hand, harmonic incongruities result in a late negativity and interfere with semantic processing; on the other hand, harmonic incongruities result in a late positivity and interfere with syntactic processing. Despite the robustness of these effects and the widespread adoption of the musical syntax or musical semantics paradigms, no satisfactory explanation has yet been offered for the existence of two different types of ERP effects elicited in response to harmonic incongruities.

In a review of neuroimaging data anchored around the Shared Syntactic Integration Resource Hypothesis (SSIRH), Patel (2008) drew upon differences in the instructions given to participants to suggest that the P600 was only elicited in situations in which participants were explicitly asked to focus their attention on the musical stimuli. A musicianship-based explanation revolves around the fact that the P600 has mostly only been observed in musicians while the N500 has been seen in non-musicians. However both explanations are faced with counter examples (e.g. Steinbeis et al., 2006).

**Figure 1:**
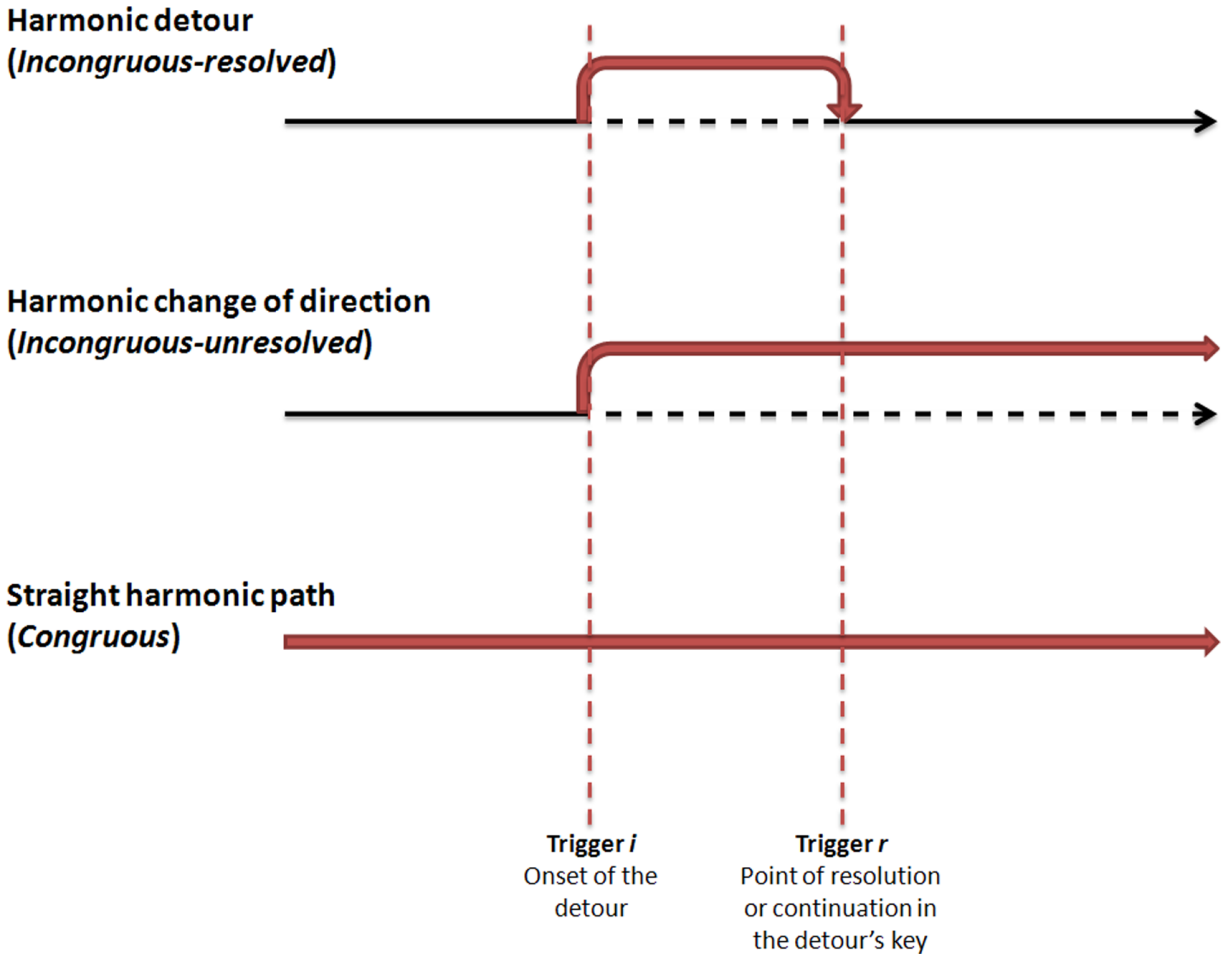
Illustration of the metaphor of harmonic detours, changes of direction and straight paths.

A closer look at the stimuli used in the studies mentioned above suggests that discrepancies in the nature of the harmonic incongruities could account for the existence of these two distinct effects in the literature. To illustrate the difference between the two main types of stimuli used to investigate harmonic incongruity processing, we will use the metaphor of following a path to a certain destination (see Figure 1). A piece which contains no harmonic incongruities can be seen as a straight path. Most pieces are not straight paths, and contain either harmonic detours (a harmonically incongruous passage which later resolves back to the original key), or harmonic changes of direction (a harmonic incongruity which leads to a permanent key change), which are typically used for aesthetic effect by composers.

The incongruous chords used in studies showing an N500 are usually chromatic chords (mostly Neapolitan 6ths). These chords, which contain notes borrowed from keys other than the main key of the piece, have been widely used by composers since the 18th century as a means of deferring the completion of a harmonic sequence when the expectation has been set for an imminent finish [35]. As such, these chords are typically used in Western tonal harmony to create harmonic detours. However, in studies using the Neapolitan chord paradigm, the chords which elicit an N500 are unorthodoxly presented as the final chord of a five-chord sequence where the tonic, or “home” chord should be [10], [13], [28], [30]. As such, these incongruities leave the expectancy violation caused by the incongruous chord unresolved: the incongruity leads to a permanent change of direction with no return to the original path. In Hantz et al.'s study [25], in which the data suggest a late negativity as a result of a violation of musical expectancies, the chords of interest were discussed not as harmonic incongruities per se (i.e. out of key chords), but as a lack of resolution or “closure” in the sequences.

In contrast, in studies reporting a P600 effect [3], [6], [8], [31]–[33], [36], or demonstrating an interaction of harmonic processing with syntactic processing [9], the incongruous chords were embedded within otherwise congruous harmonic progressions, and constituted a harmonic detour: after the incongruous chord, the chord progression returned to the original key. These observations suggest that the N500 may reflect the processing of a lack of harmonic resolution (an unresolved incongruity which leads to a permanent key change), while the P600 may reflect the processing of a harmonic detour within a harmonically resolved sequence.

### The present experiment

The processing of harmonic incongruity and of harmonic resolution have been confounded in studies to date. The present experiment addressed this for the first time by using a purpose-built stimulus set [2] in which both harmonic congruence and harmonic resolution were manipulated separately, creating three conditions: *congruous*, *incongruous-resolved* and *incongruous-unresolved*.

**Figure 2:**
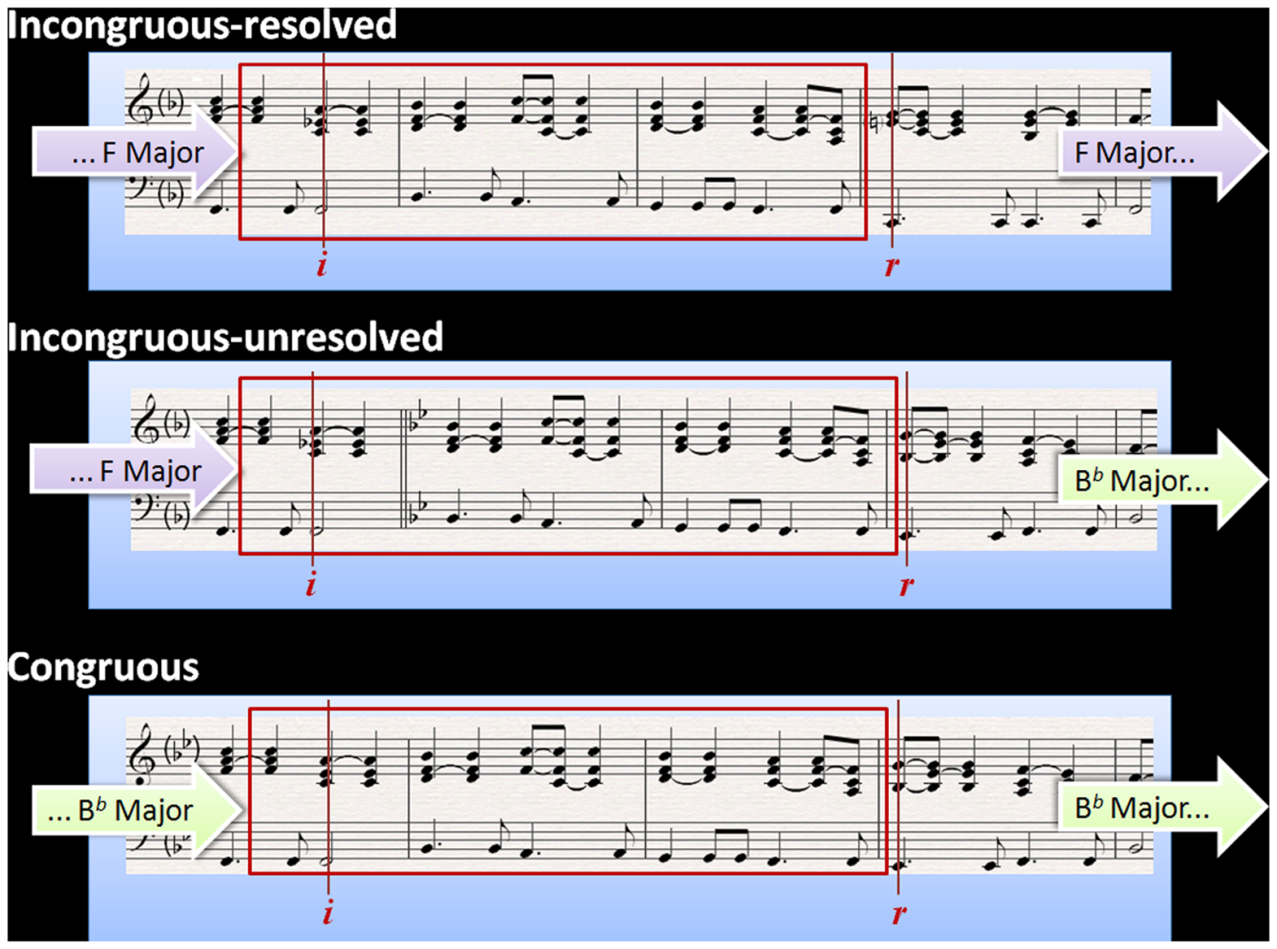
Example Harmony stimulus in all three congruity conditions. Trigger *i* marks the onset of the harmonic incongruity (or matched congruity) and trigger *r* marks the onset of the harmonic resolution (or lack of). Two quavers are kept identical either side of the chords of interest. In the example here, the incongruous conditions start in the key of F major and the harmonic incongruity is a chord of F7 (borrowed from the key of B flat major). The *incongruous-resolved* condition resolves harmonically when it returns to the original key of F major (trigger *r*) whereas the *incongruous-unresolved* condition, the equivalent chord continues in the adopted key of B flat major. In the *congruous* condition, the entire sequence is in the key of B flat major.

Participants listened to the stimuli whilst EEG was recorded. ERPs were formed time-locked to 1) harmonic incongruities: a chord that which indicated the start of a harmonic detour (trigger *i* in Figure 1), and 2) lack of harmonic resolution: a chord which confirmed a permanent change in the key (trigger *r* in Figure 1). We predicted, firstly, that harmonic incongruities would elicit a P600 in the *incongruous-* conditions compared to the *congruous* condition. Secondly, we predicted that the lack of harmonic resolution would elicit an N500 in the *incongruous-unresolved*) condition compared to the *congrous* condition (the *incongruous-resolved* condition. Considering that the P600 has mostly been reported in studies testing only musicians while the N500 has been shown in mixed groups, this experiment also compared the effects across musicians and non-musicians. To gain further insights into the relationship between these ERP patterns and listeners' impressions of the stimuli, participants also provided ratings on seven different scales pertaining to their subjective appraisal of the stimuli.

## Materials and Methods

### Participants

Twenty non musicians (15 females, mean age  = 23.00, sd  = 10.43, mean years musical training  = 0.15, sd  = 0.36) and 20 musicians (14 females, mean age  = 23.40, sd  = 9.76, mean years of musical training  = 9.77, sd  = 3.19) recruited from the University of Leeds community took part in the study. An additional 17 participants (13 non-musicians, 4 musicians) were tested and excluded after data pre-processing due to a poor signal to noise ratio in the data (see *EEG data pre-processing*). Non musicians were defined as participants with up to one year of extra-curricular musical training. Musicians were defined as participants having achieved either Grade 8 in a musical instrument or voice, and/or an A-level in music.

All participants were naive to the hypotheses and to the experimental manipulation of the stimuli. All participants were right handed, native speakers of British English, with no known language or hearing impairments, no neurological conditions, no neurological medications, and no skin conditions or wounds on their scalp.

The research was granted ethical approval by the Institute of Psychological Sciences Ethics Committee (Ref 09061–05). Informed written consent was obtained from all participants.

### Stimuli

The stimuli used in this study were from the *harmony* part of the Featherstone set [2], examples of which can be listened to at www.carafeatherstone.co.uk/research/stimuli. The set was purpose-built for the study of musical and linguistic incongruities and manipulated both congruence and resolution. The *harmony* stimuli were derived from mainstream popular music extracts containing “harmonic detours”, which can be described as a short series of notes which contains notes which do not belong to the main key of the piece. For example, in a piece for which the main key is C Major, a slight musical detour via the key of G Major can be used for effect. This would involve including a short sequence of notes featuring an F#, which is a note which does not belong in the key of C Major (the main key of the piece). Since harmonic detours contain notes from a different key to the main key of the piece, they sound slightly unusual or unexpected.

We systematically manipulated these musical extracts to create piano pieces from which the original extracts could not be recognised. There were three experimental *congruence* conditions illustrated in Figure 2.

#### Incongruous-resolved

Contains a harmonic incongruity at trigger *i* (e.g., a chord of F7, which contains an E flat, is embedded in a sequence in F Major, a key which contains E naturals, not E flats). The occurrence of the E flat creates a harmonic detour via the key of B flat Major (which contains both B flats and E flats). This detour eventually resolves by returning to the original key at trigger *r* (e.g., the Es are natural rather than flat after trigger *r*, consistent with the original key of F Major).

#### Incongruous-unresolved

Contains a harmonic incongruity at trigger *i* (e.g. the same chord of F7 containing an E flat). The harmonic detour never returns to the original key, instead it remains unresolved, confirming the new key at trigger *r* (e.g., confirming the permanent change from F Major to B flat Major).

#### Congruous

Contains no harmonic incongruities because although the chord at trigger *i* is acoustically identical across all conditions, its context has been altered to match it (e.g., the same chord of F7, which contains an E flat, is embedded in a sequence which is entirely in B flat Major which normally already contains E flats).

The ERP analyses were time-locked to trigger *i* and trigger *r* to compare neurophysiological responses between conditions at the onset of the incongruity and the point of the lack of resolution respectively. Importantly, the acoustics of the critical chords were kept identical across conditions (see Figure 2). Specifically, the harmonically congruous chord in the *congruous* condition (at trigger *i*) was acoustically identical to the incongruous chords in the *incongrouous-* conditions for which it served as the baseline. This was achieved by altering the context of the chord rather than altering the chord itself, as detailed above. Trigger *i* occurred after an average of 10.84s, allowing sufficient time for the build up of a stable tonal centre. To further ensure comparability between conditions, two quavers either side of the harmonically incongruous (or matched congruous) chord were kept identical. The chord confirming a new key in the *incongruous-unresolved* condition, which denoted a lack of harmonic resolution (at trigger *r*) was identical to the chord in the *congruous* condition, which served as its baseline.

In summary, this manipulation of the musical extracts allowed the systematic investigation of the effects of both harmonic congruence (at trigger *i*) and lack of harmonic resolution (at trigger *r*) through analysis of ERPs elicited in response to two critical chords within each musical sequence. To investigate the effect of harmonic incongruity we compared ERPs elicited at trigger *i* in response to the onset of the harmonically incongruous chord (*incongruous-resolved* and *incongruous-inresolved*) relative to an acoustically identical congruous chord (*congruous*). To investigate the lack of harmonic resolution after a period of harmonic incongruity we compared ERPs elicited at trigger *r* in response to the chord confirming the new key (*incongruous-unresolved*) to an acoustically identical chord that did not follow a period of incongruity (*congruous*).

The point at which the *incongruous-unresolved* stimuli did not resolve (trigger *r*) was closer to musical incongruities typically seen in the literature, as the chords signalling the lack of harmonic resolution featured additional notes which are not part of the original key of the piece. All twelve keys of Western Tonal harmony were equally represented in the stimuli. Chords leading to and from the chord at trigger *i* in the *congruous* condition were chosen with reference to the Table of Usual Root Progressions provided in Piston's (1978) *Harmony* [37]. The audio files were created using the standard piano sound from *Sibelius 5*'s inbuilt *KontactPlayer2* and contained no variations in dynamics or rubato. The sound files used for the three conditions were identical in the section represented by the red rectangle in Figure 2. The chord which signalled the onset of the harmonic detour occurred for the first time at trigger *i* across all conditions, to ensure that the same degree of novelty was perceived at the target point in all three conditions, and that this could be detached from the notion of incongruity. Additional design controls applied to these stimuli are discussed by Featherstone et al. (2011).

### Design and procedure

Participants were tested in a a small annexe to an EEG laboratory visible to the experimenter via CCTV. Stimuli were presented auditorily via digital stereo headphones from a personal computer running EPrime 1.2. During listening, a fixation point was provided in the form of an asterisk on the screen. Stimuli were all repeated three times in three separate blocks (A, B and C). Each participant heard 24 trials in each condition, 72 trials in total. To avoid confounding memory with congruence, participants only heard each stimulus in one condition, with an equal representation of each of the three conditions. The order of stimuli within a block was randomised between blocks and between participants.

After hearing each stimulus, each participant provided two or three ratings (3 in block A, 2 in block B and 2 in block C). These ratings, measured on a visual analogue scale in numbers of pixels from the left hand side of the scale to the participant's mouse click, captured the participant's answers to the following questions: How odd was the stimulus? (Completely normal to Very odd); How confused or perplexed do you feel, having heard to the whole stimulus? (Not at all to Very); How aesthetically pleasing was the stimulus as a whole? (Not at all to Very); In your opinion, the stimulus was... (Very bland to Very interesting); How stimulating did you find the stimulus? (Not at all to Intensely); How tense did you feel while listening to the stimulus? (Not at all to Very); How do you feel now, having listened to the whole stimulus? (Very relaxed to Very tense).

These behavioural data were collected both to ensure participants' attention was maintained on the stimuli, and to provide insight into any relationships between participants' subjective experience of the stimuli and the ERP effects elicited by the stimuli. These data were z-transformed to normalise the use of the visual analogue scale across participants. The averages per condition and per pariticant were analysed in a Musicianship (musicians vs. non-musicians) X Congruence (congruous vs. incongruous-resolved vs. incongruous-unresolved) ANOVA. Significant interactions were followed up with simple effects analyses and significant main effects were followed up with Bonferroni post hoc comparisons.

### EEG data recording

**Figure 3:**
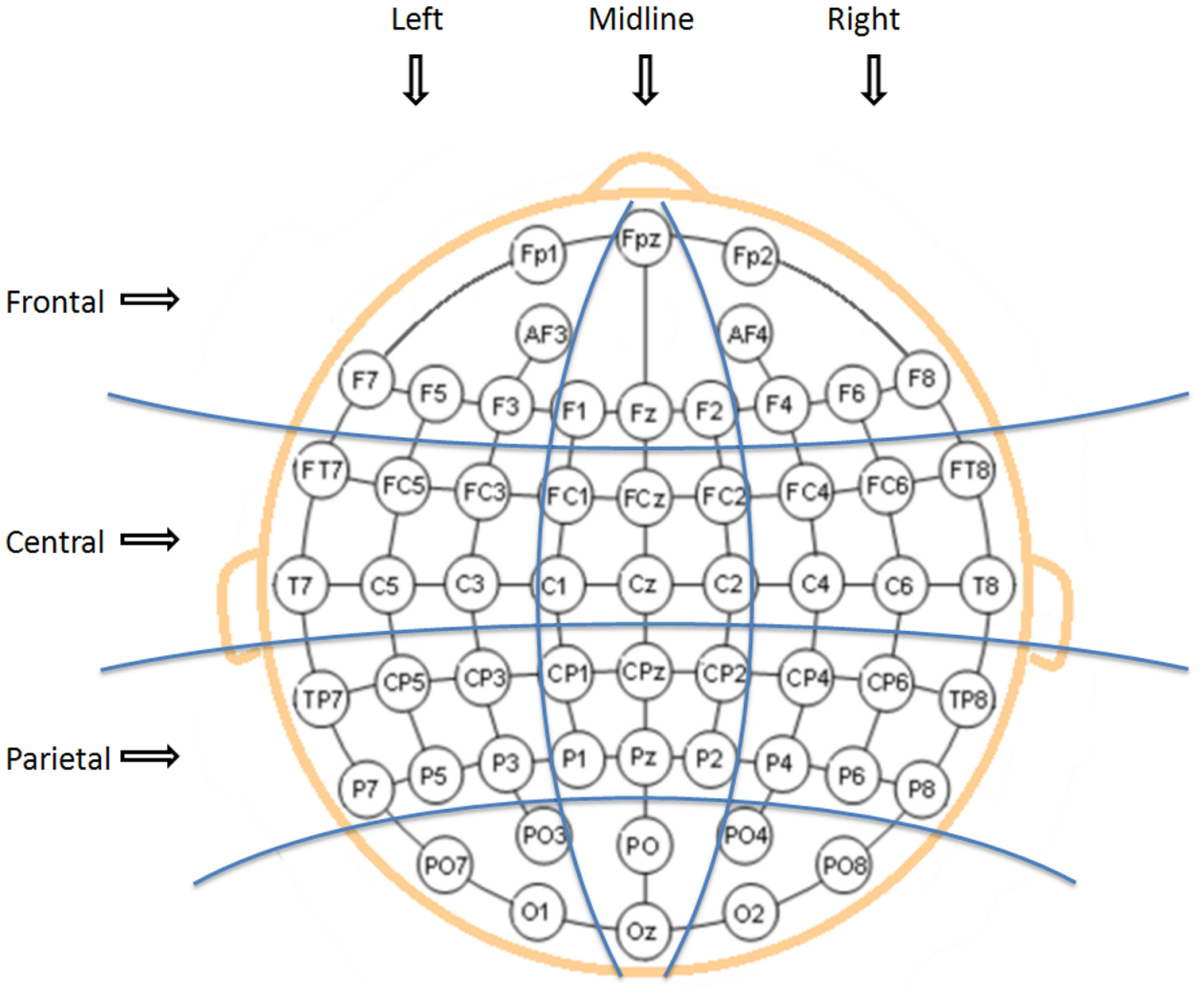
Nine electrode clusters used in the statistical analyses.

EEG was recorded using NeuroScan 4.3 Acquire and a Synamps2 amplifier from a 64-channel Ag-AgCl QuikCell cap in which electrodes were placed according to the Extended International 10–20 system (see Figure 3). Two additional electrodes were placed on the mastoids. Vertical and horizontal electro-oculograms were recorded by placing one electrode on the outer canthi of both eyes, one above and below the right eye to monitor eye movements. The ground electrode was positioned between FPz and Fz. Data were recorded using a central reference positioned between Cz and CPz. The continuous EEG data were sampled at 1000Hz and filtered online using a 200Hz low-pass filter.

### EEG data pre-processing

EEG data were analysed offline using NeuroScan 4.3 Edit software. Data were re-referenced offline to the average of the left and right mastoid electrodes. They were then band-passed filtered (0.1 to 30Hz, slope of 24dB/octave). The continuous data were visually inspected and segments were rejected if they appeared to be very noisy or saturated. Eyeblink artifacts were corrected using NeuroScan ocular artefact rejection based on a minimum of 32 blinks per participant. EEG data were epoched and ERPs formed timelocked to the onset of the incongruous chord (trigger *i*) and to the onset of the (non-) resolution chord (trigger *r*), from 100 ms before the trigger to 1300 ms after the trigger. Epochs were excluded if the amplitude exceeded +/−75 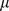
V on any channel. Participants whose data had low signal to noise ratio (fewer than 16 in any condition cell) were excluded from the analysis. In the final set of participants, the number of retained trials per condition cell was approximately 20 across all conditions. Data were smoothed over five points and baseline corrected using the pre-trigger interval (−100 ms to target chord onset onset). Epochs were averaged for each condition across all participants.

The data from the 64 electrodes were averaged into nine clusters, or *regions of interest* as shown in Figure 3: left frontal (FP1, AF3, F3, F5, F7), left central (FT7, FC5, FC3, T7, C5, C3), left parietal (TP7, CP5, CP3, P7, P5, P3), midline frontal (FPz, F1, Fz, F2), midline central (FC1, FCz, FC2, C1, Cz, C2), midline parietal (CP1, CPz, CP2, P1, Pz, P2), and right frontal (FP2, AF4, F4, F6, F8), right central (FC4, FC6, FT8, C4, C6, T8), right parietal (CP4, CP6, TP8, P4, P6, P8). Analyses were performed to investigate the effect of harmonic incongruity and its resolution (or lack thereof) on musicians and non-musicians by comparing ERPs (1) at the onset of the harmonic incongruity (trigger *i*) and (2) at the onset of the harmonic resolution/lack of (trigger *r*).

Time-windows for statistical analysis were chosen based on visual inspection of the data and previous literature. The reported amplitudes are the mean amplitude of the EEG data over the specified time-window. Data were statistically analysed using a Musicianship (musicians vs. non-musicians) X Congruence (congruous vs. incongruous-resolved vs. incongruous-unresolved) X Location (frontal vs. central vs. parietal) x Laterality (left vs. midline vs. right) ANOVA at trigger *i*. The same analysis was applied at trigger *r*, but with only two levels of Congruence (congruous vs. incongruous-unresolved), since the stimuli were only acoustically identical in these two conditions at trigger *r*. Significant interaction effects were followed up with simple effects analyses in the form of further ANOVA and planned comparisons. Only significant results involving the factors of interest are reported.

## Results

### Effects of harmonic incongruity: analysis at trigger *i*

**Figure 4:**
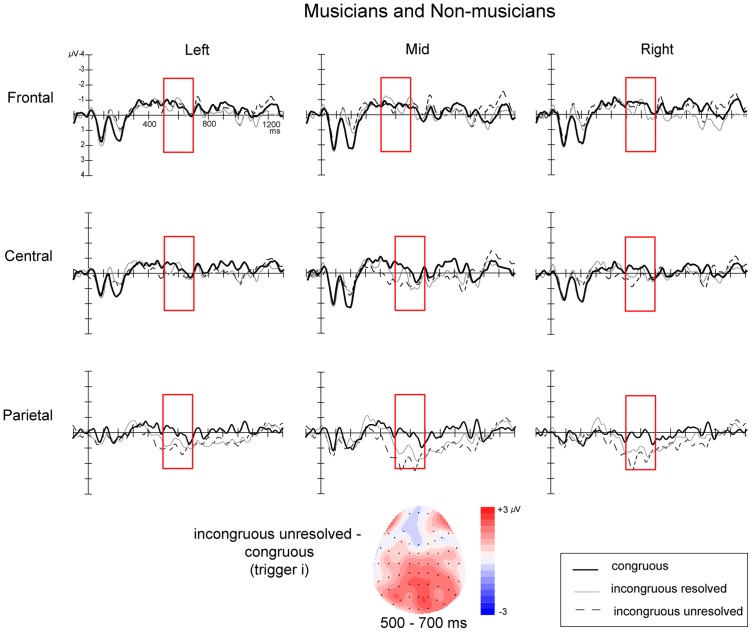
ERPs time-locked to the onset of the harmonic incongruity (trigger *i*) across all participants. The figure shows the 9 electrode clusters used in the analyses. Negative is plotted upwards. Topographic map shows the distribution of the difference between the incongruous (average of *incongruous-resolved* and *incongruous-unresolved*) and *congruous* conditions averaged over the time window of interest (500–700 ms).

ERPs time-locked to the onset of the harmonic incongruity (trigger *i*) showed a late centro-parietal positivity which was most clearly defined between 500 ms and 700 ms for the *incongruous-* conditions compared to the *congruous* condition (see Figure 4).

The initial ANOVA demonstrated a significant Congruence X Location interaction (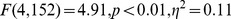). This was followed up by simple effects analyses at each location separately, which revealed an effect of Congruence for the parietal location only (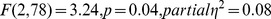), reflecting an overall posterior positivity in *incongruous-* compared to *congruous* condition across all participants.

Although there was no interaction with Musicianship in the main ANOVA, given our interest in potential between-group differences and the differences between studies involving musicians and non-musicians, we ran an ANOVA with factors of Congruence X Musicianship X Laterality, at the parietal location where effects were maximal. This revealed a significant interaction between Congruence and Musicianship (
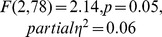
) and thus we performed further analyses at the parietal location on the two groups separately.

Non-musicians showed no significant effects but musicians showed a significant main effect of Congruence (
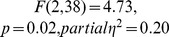
) and an interaction between Congruence and Laterality (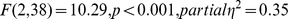). Follow-up analyses in musicians revealed significant effects of Congruence in the midline parietal cluster (
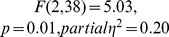
), and the right parietal cluster (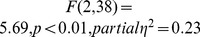). Planned contrasts in the right parietal cluster demonstrated a significant relative positivity for the *incongruous-resolved* condition (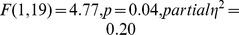) and the *incongruous-unresolved* condition (
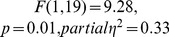
) relative to the *congruous* condition.

### Effects of harmonic resolution: analysis at trigger *r*

**Figure 5:**
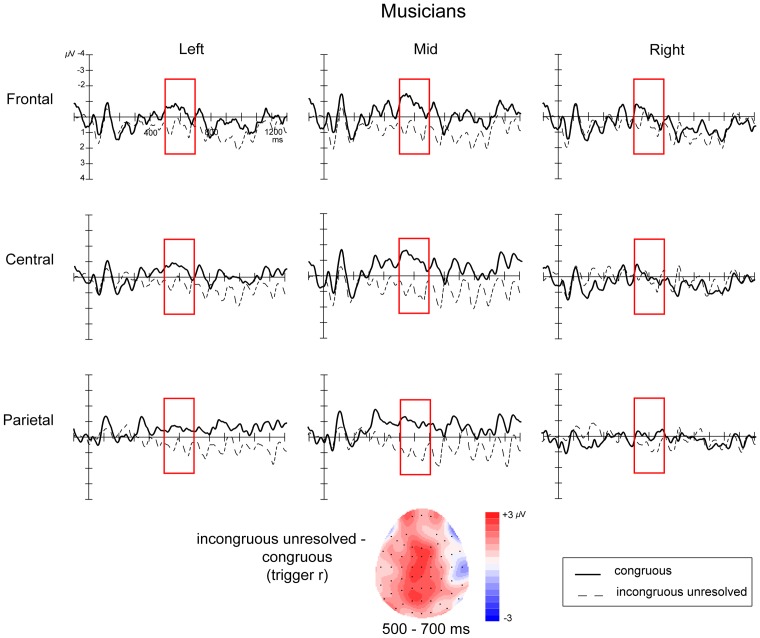
ERPs time-locked to the onset of the harmonic resolution, or lack thereof (trigger *r*) for musicians. The figure shows the 9 electrode clusters used in the analyses. Negative is plotted upwards. Topographic map the distribution of the difference between *incongruous-unresolved* and *congruous* conditions averaged over the time window of interest (500–700 ms).

**Figure 6:**
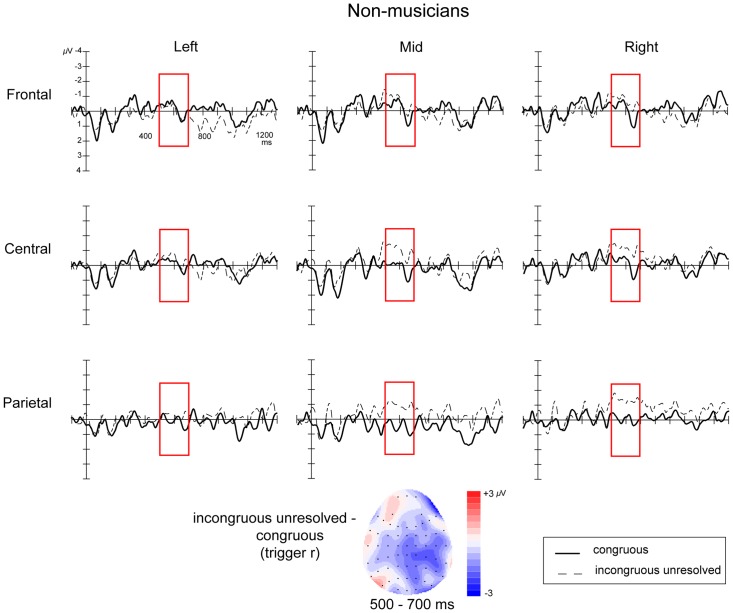
ERPs time-locked to the onset of the harmonic resolution, or lack thereof (trigger *r*) for non-musicians. The figure shows the 9 electrode clusters used in the analyses. Negative is plotted upwards. Topographic map the distribution of the difference between *incongruous-unresolved* and *congruous* conditions averaged over the time window of interest (500–700 ms).

ERPs time-locked to the onset of the lack of harmonic resolution (trigger *r*) to compare the *congruous* and *incongruous-unresolved* conditions looked very different in musicians and non-musicians (Figures 5 and 6). In musicians, the *incongruous-unresolved* condition elicited a late centro-parietal positivity at trigger *r*, relative to *congruous* condition, which was most clearly defined between 500 and 700 ms. In non-musicians, however, a late negativity was observed in the *incongruous-unresolved* condition relative to the *congruous* condition at trigger *r*, which onset around 400 ms, peaked around 570 ms, and was most clearly defined between 500 and 700 ms.

An initial ANOVA revealed an interaction between Congruence, Musicianship, and Laterality (
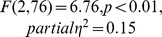
) providing strong support for between-group differences. To explore these differences further, additional analyses were performed for the two groups separately with factors of Congruence, Laterality and Location.

For musicians, there was a significant interaction of Congruence with Laterality (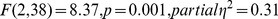). Follow-up simple effects analyses revealed that the effect of Congruence was significant only in the in the midline clusters (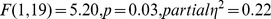), where a Location X Congruence interaction effect was also found (
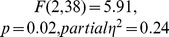
). Follow-up analyses in the midline frontal, midline central and midline parietal regions, in the form of two-tailed repeated measures t-tests revealed a significant positivity in the *incongruous-unresolved* condition compared to the *congrous* condition in the midline central (
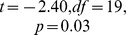
) and midline parietal clusters (
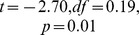
).

For non-musicians, there was a near significant Congruence X Laterality interaction effect (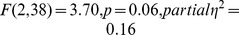). Follow-up analyses within the left, midline and right clusters revealed a significant main effect of Congruence within the right clusters (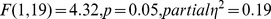), demonstrating a significant negativity in the *incongruous-unresolved* condition compared to the *congruous* condition.

### Behavioural data

**Figure 7:**
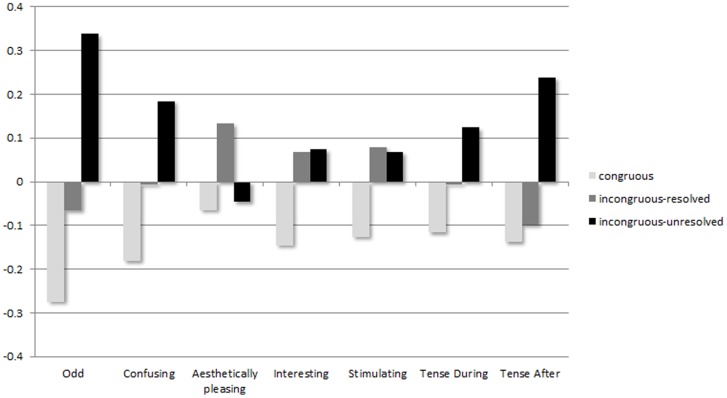
Mean z-scores rating per condition on each visual analogue scale. The negative end of the scale (“not at all”, “completely normal”) is towards the negative end of the y-axis; the positive end of the scale (“very”, “intensely”) is towards the positive end of the y-axis.

The trends displayed by the mean ratings in each of these scales, displayed in Figure 7, suggested that the *Incongruous-unresolved* stimuli were on average considered more odd, confusing and tension-inducing than *incongrous-resolved* stimuli, which, in turn were more odd, confusing and tension inducing than Congruous stimuli. These data also suggested that harmonic incongruities led to musical stimuli being rated more as more interesting and more stimulating than congruous stimuli, regardless of whether the incongruities resolved. However, stimuli were only rated as more aesthetically pleasing than congruous stimuli when incongruities subsequently resolved (*incongruous-resolved* condition).

Table 1 displays the outcomes of the Musicianship (musicians vs. non-musicians) X Congruence (congruous vs. incongruous-resolved vs. incongruous-unresolved) ANOVA carried out on each rating scale. Of particular interest to this study investigating ERP effects associated with the processing of incongruities were the “how confusing” and “how odd” scales. The “how confusing” scale showed a significant main effect for Congruence (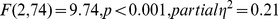) and no significant Congruence X Musicianship interaction, reflecting the fact that, regardless of musicianship, participants perceived the *incongruous-unresolved* stimuli to be significantly more confusing than *congruous* stimuli. Participants across both musicianship groups also reported feeling significantly more tense after the listening stimuli in the *incongruous-unresolved* condition than after listening to *congruous* and *incongruous-resolved* stimuli (main effect: 
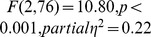
). The “how odd” scale showed both a significant main effect for Congruence (
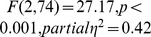
) and a significant Musicianship X Congruence interaction (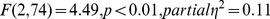). This interaction was followed up by a simple effects analysis, splitting the data by Musicianship. These analyses revealed a significant effect of Congruence on oddity ratings in both musicians (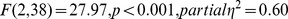) and non-musicians (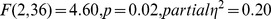). Bonferroni post hoc comparisons showed that while both groups rated the *incongruous-unresolved* stimuli to be significantly more odd than the *congruous* stimuli (musicians, 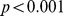; non-musicians, 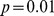), only musicians rated the *incongruous-resolved* stimuli to be more odd than the *congruous* stimuli (musicians, 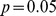; non-musicians, 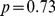). Despite this between-groups difference in the conscious perception of how odd the *incongruous-resolved* stimuli were relative to the *congruous* stimuli, the “how interesting” scale showed a main effect of Congruence (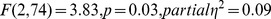) but no Musicianship X Congruence interaction effect: across both groups of participants both the *incongruous-resolved* and the *incongruous-unresolved* stimuli were rated as significantly more interesting than the *congruous* stimuli.

**Table 1:** Outcome of the Musicianship X Congruence ANOVA carried out on the data from each of the visual analogue scales.

Scale	Main effect of congruence	Bonferroni post hoc comparisons	Congruence X Musicianship interaction
Odd	* 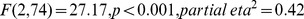	The comparisons showed different patterns in musicians and non-musicians.	* 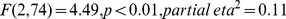
Confusing	* 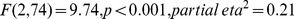	Incongruous-Unresolved was significantly more confusing than Congruous. No other differences were significant.	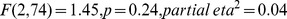
Aesthetically pleasing	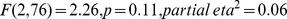	–	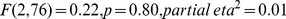
Interesting	* 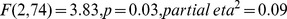	There was no significant difference between the two Incongruous conditions; they were both significantly more interesting than Congruous.	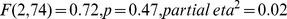
Stimulating	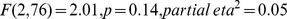	–	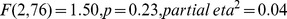
Tense During	* 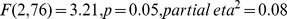	No significant differences were found in the pairwise comparisons.	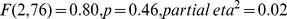
Tense After	* 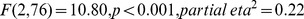	Participants felt significantly more tense after Incongruous-Unresolved than after the two other conditions; there was no significant difference between Congruous and Incongruous-Resolved.	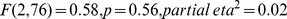

(* indicates a statistically significant effect).

### Summary of results

These analyses demonstrated firstly that harmonic incongruities (trigger *i*) elicited a significant late centro-parietal positivity across both participant groups in the *incongruous-* conditions compared to the *congruous*. Subsequent analyses indicated that the effect was driven by an effect in musicians only, who alone found the *incongruous-resolved* stimuli significantly more odd than the *congruous* stimuli. Despite these between groups differences in ERP patterns and oddity ratings, stimuli were found to be significantly more interesting in the *incongruous-resolved* condition compared to the *congruous* condition across both participant groups. Secondly, in response to the lack of harmonic resolution (trigger *r* in the *incongruous-unresolved* condition) there was a further significant late centro-parietal positivity in musicians and a near significant late right negativity in non-musicians compared to the *congruous* condition. Stimuli in the *incongruous-unresolved* were also found to be significantly more confusing, more odd, more interesting and more tension-inducing than the *congruous* stimuli across all participants.

## Discussion

Using a purpose-built stimulus set [2] we investigated the effects of both harmonic incongruity and harmonic resolution, which have been confounded in previous studies. We recorded the electrophysiological brain responses of musicians and non-musicians while they listened to musical excerpts and formed ERPs time-locked to chords which denoted (1) the onset of a harmonic incongruity and (2) the lack of harmonic resolution of the musical piece, as well as rating data indicating the participants' subjective appraisal of the stimuli.

### Harmonic incongruity

Harmonically incongruous chords resulted in a late posterior positivity, strongest between 500 ms and 700 ms post-chord onset, which was similar in timing and distribution to the effect reported in Patel et al.'s (1998) seminal study as a P600. In line with other studies reporting a late positivity in response to harmonic incongruities [31], [32], [34], we suggest the positivity reflects the efforts involved in integrating harmonic incongruities into their context.

Studies reporting a late positivity have typically only tested musicians [3], [31], [32] or found stronger positivities in musicians than in non-musicians [8], [34]. Although we found no significant interaction between Musicianship and Congruence, simple effects analyses investigating the groups separately showed that the ERP effect was significant only in musicians. This result mirrors the findings of previous studies, and perhaps suggests a larger or more consistent effect for musicians. Such claims must of course be treated with caution and would require statistical support from future studies.

The behavioural data in the *incongruous-resolved* condition revealed similarities between musicians and non-musicians in how interesting the stimuli were perceived to be, but differences in how odd they were perceived to be. The trend in the “aesthetically pleasing” ratings seemed to lend support to theories building on the work of Meyer [38] claiming that it is not the incongruity or musically unexpected element per se but its resolution that leads to aesthetic pleasure. The ANOVA revealed that this condition was found to be significantly more “interesting” than the *congruous* condition across both participant groups. However, only the musicians rated this condition as significantly more “odd” than the *congruous* condition. This suggests that the the P600 could be related to consciously perceiving a specific element as being incongruous with its context: non-musicians may have perceived these stimuli to be slightly out of the ordinary but without knowing exactly why. Note that this condition was not found to be significantly more “confusing” than the *congruous* condition, further suggesting that the incongruity, although perceived as “odd” by musicians, was successfully integrated into its context.

### Lack of harmonic resolution

The lack of resolution of the harmonic detour resulted in different ERP patterns in musicians and non-musicians. In musicians, the onset of the chord at trigger *r* in the *incongruous-unresolved* condition, which marked a permanent change in harmonic direction, elicited a significant positivity in comparison to the *congruous* baseline. This effect was very similar in timing and topography to the positivity elicited by the harmonic incongruity at trigger *i*, suggesting that both these chords were processed in a similar way by musicians. By contrast, in non-musicians, the lack of resolution resulted in a late negativity beginning around 400 ms post-chord onset, peaking around 570 ms, which was significant over right scalp regions.

The negativity seen in non-musicians was similar in timing to negativities reported in previous studies as an N500 [5], [27]. Although the significance of this effect fell just short of the 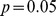 benchmark for statistical significance (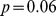 for the interaction of Congruence with Laterality, 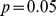 for the effect of Congruence within the right regions), the effect sizes (respectively 0.16 and 0.19) exceeded the 0.14 value considered as the threshold for “large effects” when using the partial eta squared calculation of effect size [39]. We also note that a previous study emphasised the sometimes elusive nature of late negativities in response to subtle harmonic incongruities [40]. The distribution of the effect was less anteriorly focussed than previous observations [7], which may reflect differences in the rhythmic patterns used in the stimuli. In particular, the stimuli typically used in studies reporting an N500 (e.g. [5], [27], [28]) consist of chorale-like sequences, in which a number of chords are played at equal intervals until the final chord of the sequence is played. The stimuli in the present study were designed to sound more like natural pieces of piano music. This was achieved, in part, by the inclusion of rhythmically rich patterns. The difference in rhythmic information between the constant durations in previous chorale-type stimuli (near null) and the rhythmically variable patterns in the stimuli used in this study (rich) is a plausible candidate for distributional differences. While this explanation remains in need of further research, it has some support from studies on rhythmic processing that elicited widely distributed ERP effects [41].

The differences between the response of musicians and non-musicians to the lack of harmonic resolution (trigger *r*) could be accounted for by the way in which musicians and non-musicians attend to music. Musicians, whose training, according to the OPERA hypothesis [42], requires and nurtures the development of focused attention, have been shown to have a more local and analytical approach to music processing than non-musicians, who have a more holistic approach [42]–[45]. A more focussed and local approach to music processing means that the chord precluding harmonic resolution could have been perceived by musicians as a new incongruous element, similar to the first incongruous element. If indeed musicians had already successfully integrated the first harmonic incongruity (e.g. a chord belonging to the key of B flat Major) into the original key of the piece (e.g. F Major), as suggested by the presence of the P600 and the behavioural data in the *incongruous-resolved* condition, then the chord precluding harmonic resolution at trigger *r* in the *incongruous-unresolved* condition (e.g. a chord repeating a note from B flat Major) would merely be perceived as a new harmonic detour, to be treated in the same way as the original harmonic incongruity (trigger *i*).

An alternative interpretation of this P600, in line with the processing of garden path sentences, could be that upon encountering trigger *r* musicians reinterpret the section between trigger *i* and trigger *r* as belonging to the confirmed new key (e.g. B flat Major). We are grateful to an anonymous reviewer for this interpretation of the effect at trigger *r*. By contrast, non-musicians, who favour a more holistic approach, may perceive the chord at trigger *r* as a definitive step away from the original key of the piece, which results in the piece sounding unfinished and leads to the stimuli being rated as significantly more odd, confusing, and tension-inducing than the *congruous* stimuli.

### Semantics, syntax or neither?

The current findings, which suggest that the late negativity is associated with a lack of harmonic resolution rather than with harmonic incongruity per se, are in line with our observations of the stimuli and associated ERP patterns reported in the literature [5], [10], [25], [27], [28]. Taking lack of resolution as the key feature in the interpretation of the ERP components, parallels can be found in linguistic processing conditions in which negativities have also been observed. For example, a sentence such as “He butters his bread with his...” leaves the participant expecting the word “knife” to complete the picture and bring closure or resolution to the message. By swapping the word “knife” for “socks” the picture is not complete, but instead creates an expectation for the opening of a different path in the narrative that would offer an explanation for the odd behaviour described, or result in a revaluation of the mental representation built up so far. Similarly, the introduction of a harmonic detour (incongruous-resolved) in an otherwise harmonically congruous sequence echoes the introduction of a relative clause in a well-formed and meaningful sentence. The harmonic detour requires establishing harmonic relationships between the original key and the key of the modulation, while the syntactic detour requires keeping track of dependencies.

This conceptualisation of “resolution” across music and language provides a plausible basis for the different interferences between harmony and language processing in the literature. The interpretation of the N500 as being elicited by the detection of a lack of harmonic resolution helps makes sense of the observed interference of lexico-semantic processing of language on late negativities elicited by unresolved chord sequences [7], [10], [30]. Steinbeis and Koelsch (2008) who used “direction changing” (incongruous-unresolved) harmonic incongruities, which elicited a late negativity in this study, reported an interaction with the processing of semantic incongruities, which also typically elicit a late negativity, but not with syntactic incongruities. The interpretation of the P600 as reflecting processes of local integration also helps make sense of the interactions between musical “detours” and the processing of complex embedded syntactic clauses in language. Slevc et al. (2009) who used “detour” (incongruous-resolved) harmonic patterns, which elicited a late positivity in the present study, reported an interaction with the processing of syntactic incongruities, which also typically elicit a late positivity.

From these observations, it would seem that the way in which harmonic processing interacts with language processing has to do with the resolved or unresolved nature of the harmonic incongruities. To test this explanation of the different patterns observed in previous studies, future research should investigate how the processing of harmonic incongruity and harmonic resolution interact with the processing of semantic and syntactic incongruities. This could be accomplished using a method similar to that used by Slevc et al. (2009) and Steinbeis and Koelsch (2008), by manipulating which types of incongruities occurred concurrently: pairing either semantic or syntactic incongruities with either resolved or unresolved harmonic incongruities.

## Conclusion

Using electrophysiological brain responses recorded while participants listened to short musical pieces, we showed that harmonically incongruous notes or chords embedded within an otherwise harmonically congruous sequence (*incongruous-resolved*) elicited a late centro-parietal positivity, similar to the P600 originally reported by Patel et al. (1998). At the point where the harmonic incongruity failed to resolve back to the original key of the piece (*incongruous-unresolved*), responses differed depending on the musicianship of the listeners. For musicians there was another positivity similar to the P600; for non-musicians there was a late negativity similar in time-course to the N500 but with a wider distribution. We suggest that the differences between musicians and non-musicians in response to the chord precluding harmonic resolution can be explained by the listening style of the two groups of listeners. The behavioural data collected alongide the EEG data suggested that the P600 may be associated with a more conscious and analytic perception of an element as being incongruous with its immediate context, while the N500 may reflect a more general confusion- and tension-inducing sense of lack of resolution resulting from a more holistic listening style. These results pave the way for more investigations into the effects of musical training on harmonic processing, into the effects of other stimulus characteristics (rhythm, voicing of incongruities) on the distribution of ERP effects, and into interference effects between music and language processing.

This is the first study to provide empirical evidence to account for the existence of two different late ERP responses to harmonic incongruities [3], [7]. Its findings emphasise the importance of considering the characteristics of both the stimuli and the listeners in establishing the functional significance of music- and language-elicited ERP effects. By introducing the notion of resolution into the discussion of harmonic processing, this study makes sense of the apparent contradiction between studies which have made the case for equating harmony with musical semantics and those which have presented harmony as musical syntax: if the differences in ERP effects between these studies can be accounted for by the resolved or unresolved nature of harmonic incongruities, harmony does not need to be equated with either. The case for resolution instead emphasises the similarities in pattern processing and incongruity integration across these two domains of human cognition.
